# Willingness to accept long-acting injectable antiretroviral therapy among persons living with HIV in a tertiary facility in Southern Ghana: Key consideration for programming

**DOI:** 10.1371/journal.pgph.0005984

**Published:** 2026-06-26

**Authors:** Peter Puplampu, Vincent Ganu, Isaac Kyeremateng, Emmanuella Ayinbisa Amankwa, Oluwakemi Oladele, Francisca Zigah, Kofi Agyabeng

**Affiliations:** 1 Department of Medicine and Therapeutics, Korle Bu Teaching Hospital, Accra, Ghana; 2 Department of Internal Medicine, University of Ghana Medical School, Accra, Ghana; 3 School of Public Health, University of Ghana, Accra, Ghana; 4 Institute of Informatics, Data science and Biostatistics, Washington University School of Medicine, St Louis, Missouri, United States of America; 5 Department of Psychiatry, Korle Bu Teaching Hospital, Accra, Ghana; 6 Department of Pharmacy, Korle Bu Teaching Hospital, Accra, Ghana; 7 Department of Biostatistics, School of Global Public Health, New York University, New York, New York, United States of America; London School of Hygiene and Tropical Medicine, UNITED KINGDOM OF GREAT BRITAIN AND NORTHERN IRELAND

## Abstract

There a gradual transformation of the HIV epidemic with the emergence of antiretroviral therapy. Adherence to daily pill intake is vital for optimal HIV viral suppression. A strategic approach to enhancing adherence is the development of long-acting injectable ART (LAI-ART). Patients’ willingness to accept LAI-ART is a key determinant to the success of these novel therapies, but data is lacking in Africa and Ghana. This study assessed the willingness to accept LAI-ART among patients attending HIV clinic in Accra, Ghana. A cross-sectional study was conducted among 309 patients attending HIV clinic at the Infectious Disease Center of the Korle-Bu Teaching Hospital from July to October 2023. Data on perspectives on LAI-ART was collected using a structured questionnaire. Willingness was measured after exposure to a structured educational session to increase awareness or knowledge on LAI-ART. Descriptive statistics was used to summarize the demographic and clinical characteristics. Bivariate and multivariate analysis was performed with Fisher’s exact or Chi square test and logistic regression respectively. Most (71.5%) participants were female, above 50 years (46.3%) and were employed (70.6%). Majority were on a single-pill regimen (80.2%), adherent (86.7%), and had no prior awareness about LAI-ART (69.9%). After structured educational session, 89.6% showed a preference for LAI-ART, with a preferred frequency of injections every 6 months (67.3%). Almost 50% did not prefer self-administration of LAI- ART. Thirty-two percent had concerns about LAI-ART, and about 72% of PLHIV were willing to pay for LAI-ART. Participants’ willingness to accept LAI-ART reflected a hypothetical or informed preference and potential acceptability rather than observed behavior due to structured educational session. More education on LAI-ART is needed. LAI- ART presents an opportunity to increase access and adherence to HIV treatment. Further studies assessing potential barriers and how to address them will aid in the success of this treatment regimen.

## Introduction

The HIV/AIDS epidemic has been one of the biggest threats to global health since the identification of the virus in 1981. Globally, an estimated 40.8 million people are living with HIV and over 630,000 annual deaths reported in 2024 [1]. At least 65% of people living with HIV reside in Sub Saharan Africa which continues to bear a disproportionate burden of the epidemic, accounting for approximately 380,000 HIV-related deaths annually [1,2]. In Ghana, national estimates indicate that over 15,000 new HIV infections and approximately 12,600 AIDS-related deaths occur annually, with an estimated 334,721 people currently living with HIV, the majority of whom are female [3]. Over the last two decades, the development of highly active combination antiretroviral therapy has been the standard treatment for HIV infection. Oral combination antiretroviral therapy (cART) typically consists of a combination of two nucleoside reverse transcriptase inhibitors (NRTIs) along with one nonnucleoside reverse transcriptase inhibitor (NNRTI), protease inhibitor, or integrase strand transfer inhibitor (INSTI). These regimens are taken daily to suppress viral replication, improve CD4 counts and prevent disease progression [2,4].

Oral cART has transformed the HIV epidemic by delaying disease progression, reducing sexual transmission, simplifying complex regimens to single fixed-dose combinations and improving longevity. Consequently, HIV has now transitioned from a fatal illness to a manageable chronic condition among many people living with HIV(PLHIV) [1,2]. Despite these advances, ART remains a lifelong commitment, and daily pill intake continues to pose adherence challenges for many patients. Even with reduced pill burden, factors such as stigma, pill fatigue, mental health challenges, and structural barriers affect adherence and compromise viral suppression, hindering progress toward the UNAIDS 95-95-95 targets [2,5].

A strategic approach to optimize adherence and preventing antiretroviral resistance is the development of LAI-ART. They offer an opportunity to overcome barriers associated with daily oral therapy, including the psychological and social burdens. Additional benefitsinclude simplified dosing schedules, reduced treatment fatigue, improved quality of life, and/ enhanced mental well-being. Long-acting formulations may be particularly beneficial for specific populations such as frequent travelers and individuals experiencing challenges with daily medication adherence [5,6].

Among the most advanced LAI-ART options are injectable formulations of an integrase inhibitor cabotegravir, and the non-nucleoside reverse transcriptase inhibitor rilpivirine. This combination has been approved for HIV treatment in the United States, Canada, the European Union, and the United Kingdom for individuals with sustained viral suppression [6]. Another promising long-acting agent is lenacapavir, the first capsid inhibitor for HIV treatment, offering dosing intervals of up to six months and representing a significant advancement in HIV therapeutics [7].By reducing the daily pill burden to as few as six to twelve injections per year, LAI-ART have the potential to substantially transform HIV care [5]. Although LAI-ART awareness remains limited, studies have demonstrated high patient acceptability, with more than half of PLHIV preferring injectable therapy over daily oral regimens [8]. Additionally, PLHIV who experience adherence difficulties with oral ART have expressed greater interest in LAI-ART [9].

While patient willingness to accept LAI-ART is critical to its successful uptake, acceptability alone does not guarantee feasibility within resource-constrained health systems. In Ghana, HIV care is increasingly delivered through differentiated service delivery (DSD) models that aim to improve retention and reduce the burden on both patients and health facilities through decentralized and patient-centered approaches. Integrating LAI-ART into these existing service delivery structures may offer opportunities to enhance adherence and treatment satisfaction, but it also raises important implementation considerations, including cold-chain requirements, clinic capacity, appointment scheduling, and healthcare workforce readiness. Understanding patient acceptability within this broader implementation context is therefore essential for informing national ART programming and guiding evidence-based policy decisions regarding the future integration of LAI-ART into Ghana’s HIV treatment landscape. Therefore, this study sought to determine patients’ knowledge, willingness to accept LAI-ART and factors influencing willingness to accept emerging LAI-ART among PLHIV attending HIV clinic at the Infectious Disease Center of the Korle-Bu Teaching Hospital.

## Methods

### Study setting and design

A single centre hospital-based cross-sectional study with an embedded educational exposure and thus, a pre-post data collection approach was conducted at the Infectious Disease Center of the Korle-Bu Teaching Hospital among patients receiving care at the HIV clinic.

The Korle Bu Teaching Hospital (KBTH) is a tertiary hospital in Accra, the capital city of Ghana. It is the leading national referral center in Ghana. The Infectious Disease Center at the Korle-Bu Teaching Hospital houses the largest HIV clinic in the country with over 6834 (6379 adults and 455 children) currently active on treatment.

### Study population

The study population included PLHIV aged 18 and above, attending the HIV clinic at the Infectious Disease Unit of the Korle-Bu Teaching Hospital from July 2023 to October 2023. Patients were recruited at the out-patient department (OPD) of the Infectious Disease Center of the KBTH through a simple random sampling by trained research assistants after obtaining a written informed consent. A sampling frame was created using the clinic’s ART register, which contained a list of all eligible PLHIV receiving care during the study period. Each eligible patient was assigned a unique identification number prior to the start of the clinic. Using the lottery method, participants were then randomly selected from the list until the required sample size was achieved. This approach ensured that every eligible patient had an equal chance of being selected, thereby minimizing selection bias and improving the representativeness of the sample.

### Data collection methods

A structured questionnaire with no personal identifiers was used, and data was collected electronically from consenting patients by research assistants in a private space at the clinic. The questionnaire was adapted from existing studies and was piloted at the Greater Accra Regional hospital ART clinic. The questionnaire was primarily administered in English. Trained research assistants explained questions in the local dialect for any participant who could not fully comprehend the English language for questionnaire completion. All patients were assigned a unique study ID and data collected included socio-demographic such as age, sex, employment status, marital status, and education level. Clinical data collected include years of diagnosis, number of years on ART, current ART regimen, number of pills taken per day and whether patients missed an ART pill in the past week. The last section of the questionnaire first assessed participants for baseline knowledge of LAI-ART and subsequently provided these participants with educational information about the therapy before reassessing perceptions and willingness to accept and it. After the pre-existing knowledge of participants were assessed, public health nurses provided a 45-minute structured verbal education session on LAI-ART. The education included the types of LAI-ART, their routes of administration, benefits, cost, and complications. After this education, the perceptions and willingness to accept LAI-ART among participants were reassessed.

The questionnaire administration was done by trained research assistants and collected data were password protected and accessible only by investigators. Despite the efforts at minimizing bias in the study, there may still be some potential sources of bias, including social desirability bias, participation bias and information framing effects.

### Statistical analysis

The data was imported into excel and exported into Stata version 20 for analysis. Descriptive analysis of baseline characteristics of patients was conducted and presented in appropriate frequency tables.

Associations between socio-demographic variables, clinical characteristics, current ART regimen and perceptions on LAI-ART and acceptability of LAI-ART were evaluated using Fishers exact or chi-square tests pre- and post-educational exposure. It is acknowledged that no causal inference can be drawn regarding the effect of the education A binary logistic regression model was used to determine the factors significantly associated with the acceptability of LAI-ART, reporting the crude and adjusted odds ratios of the various characteristics. Multicollinearity among independent variables was assessed and no evidence of significant multicollinearity was observed. In addition, the model goodness-of-fit was evaluated and indicated that the model adequately fit the data, supporting the reliability of the reported associations. Statistical significance was set at 95% confidence level (p-value < 0.05).

### Ethical considerations

Ethical approval was obtained from the Institutional Review Board of the Korle-Bu Teaching Hospital with approval number KBTH-STC/IRB/00002/2023. The investigators ensured strict confidentiality and anonymity throughout the study.

## Results

### Socio-demographic characteristics of participants

A total of 342 PLHIV were approached for the study with 309 consenting to participate giving a response rate of 90.4%. Most (71.5%) of the study participants were female, with the predominant age group being ≥ 50 years (46.3%). About half (49.8%) of the participants had basic education, and the majority (70.6%) were employed (Table 1).

**Table 1:** Socio-demographic and clinical characteristics of adult PLHIV accessing ART services at a tertiary facility in Accra, Ghana, 2023.

Characteristics	LAI-ART Preference		Total	P-value
	Yes n (%)†	No n (%)†	n=309 (%)‡	
Mean(SD) age, years 45(14.8)				0.681
Age groups				
18 – 24 years	38 (86.4)	6 (13.6)	44 (14.2)	
25 – 49 years	109 (89.3)	13 (10.7)	122 (39.5)	
≥ 50 years	130 (90.9)	13 (9.1)	143 (46.3)	
Sex				0.382
Male	81 (92.0)	7 (8.0)	88 (28.5)	
Female	196 (88.7)	2511.3)	221 (71.5)	
Marital status				0.319
Single	111 (92.5)	9 (7.5)	120 (38.8)	
Married/ Cohabiting	103 (86.6)	16 (13.4)	119 (38.5)	
Divorced/ Widowed	63 (90.0)	7 (10.0)	70 (22.7)	
Educational status				0.456
No formal education	47 (94.0)	3 (6.0)	50 (16.2)	
Primary	134 (87.0)	20 (13.0)	154 (49.8)	
Secondary	59 (90.8)	6 (9.2)	65 (21.0)	
Tertiary	37 (92.5)	3 (7.5)	40 (12.9)	
Employment status				0.796
Employed	197 (90.4)	21 (9.6)	218 (70.6)	
Unemployed/Retired	56 (87.5)	8 (12.5)	64 (20.7)	
Student	24 (88.9)	3 (11.1)	27 (8.7)	
Years on ART				0.833
Less than 5 years	4990.7)	5 (9.3)	54 (17.5)	
5 – 10 years	105 (90.5)	11 (9.5)	116 (37.5)	
More than 10 years	123 (88.5)	16 (11.5)	139 (45.0)	
Pills taken per day				0.015**
One pill	217 (87.6)	31 (12.4)	248 (80.2)	
More than one pill	58 (98.3)	1 (1.7)	59 (19.1)	
Ever missed pills in the past 2 weeks				0.334
Yes	35 (85.4)	6 (14.6)	41 (13.3)	
No	242 (90.3)	26 (9.7)	268 (86.7)	
Comorbidities				0.504
Yes	96 (88.1)	13 (11.9)	109 (35.3)	
No	181 (90.5)	19 (9.5)	200 (64.7)	

†-row%, ‡- col %, **- significant at p < 0.05.

### Clinical characteristics of participants

About eight in every ten (80.2%) sampled patients reported taking one pill a day, with most (86.7%) of them never missing a pill in two (2) weeks. About 35% of participants reported having a comorbid condition (Table 1).

### Awareness and preference of LAI-ART (Before and After Education) among PLHIV

Before education, only 30.1% of patients had awareness about LAI-ART with the hospital/clinic (19.7%) being the main source of information. However, after education, 89.6% were certain and willing to take LAI-ART. Most patients (67.3%) preferred a 6 monthly injection, and 72.2% willing to pay for the LAI-ART, with 68.0% expressing no fears at all about the therapy (Table 2).

**Table 2:** Awareness and preference of LAI-ART among adult PLHIV at a tertiary facility in Accra, Ghana, 2023.

Characteristics	N	%
Before education		
Awareness of LAI-ART (n = 309)		
Yes	93	(30.1)
No	216	(69.9)
Source of information (n = 93) – multiple response		
Hospital/Clinic	61	(65.6)
Friends	14	(15.1)
Radio	3	(3.2)
Social media	5	(5.4)
Television	1	(1.0)
Others*	9	(9.7)
After education		
Preference for LAI-ART after education		
Yes	277	(89.6)
No	32	(10.4)
Preferred frequency of injections		
Once every 6 months	208	(67.3)
Once every 3 months	61	(19.7)
Once every 2 months	28	(9.1)
Once a month	12	(3.9)
Preference for a Monthly or Bimonthly injection		
Once every 2 months	252	(81.6)
Once every month	26	(8.4)
Uncertain	31	(10.0)
Preference for who to administer injection		
Trained nurse	244	(79.0)
A trained volunteer	65	(21.0)
Preference for self-administered injection if trained		
No	153	(49.5)
Yes	127	(41.1)
Uncertain	29	(9.4)
Patient’s willingness to pay for LAI-ART		
Yes	223	(72.2)
No	86	(27.8)
Having fears about the use of LAI		
Yes	99	(32.0)
No	210	(68.0)

*Others included close family members, Civil Society Organizations, internet.

### Factors associated with willingness to accept LAI-ART

Participants who take one pill of ART a day had a substantially lower likelihood of willingness to accept LAI-ART compared to those who took more than one pill. (aOR: 0.1, 95% CI: 0.0-0.9) (Table 3).

**Table 3:** Factors associated with willingness to accept LAI-ART among adult PLHIV at a tertiary facility in Accra, Ghana, 2023.

Factors	cOR^&^ (95% CI)^#^	P-value	aOR^$^ (95% CI)	P-value
Age groups				
≥ 50 years	1		1	
18 – 24 years	0.6 (0.2–1.8)	0.386	0.3 (0.0–1.6)	0.141
25 – 49 years	1.4 (0.5–3.7)	0.670	0.8 (0.3–2.0)	0.187
Sex				
Male	1		1	
Female	0.7 (0.3–1.6)	0.384	0.7 (0.2–1.8)	0.437
Marital status				
Single	1		1	
Married/ Cohabiting	0.5 (0.2–1.2)	0.138	0.3 (0.1–1.0)	0.053
Divorced/ Widowed	0.7 (0.3–2.0)	0.551	0.5 (0.1–1.8)	0.263
Educational status				
No formal education	1		1	
Basic to Junior High	0.4 (0.1–1.5)	0.186	0.5 (0.1–1.7)	0.236
Senior high/vocational	0.9 (0.1–2.6)	0.526	0.7 (0.2–3.6)	0.717
Tertiary	0.8 (0.2–4.1)	0.777	1.0 (0.2–6.0)	0.994
Employment status				
Employed	1		1	
Unemployed/Retired	0.7 (0.3–1.8)	0.808	0.7 (0.1–5.3)	0.766
Student	0.9 (0.2–3.0)	0.508	0.7 (0.3–1.7)	0.424
Years on ART				
Less than 5 years	1		1	
5 – 10 years	1.0 (0.3–3.0)	0.963	0.9 (0.3–3.1)	0.887
More than 10 years	0.8 (0.3–2.3)	0.653	0.9 (0.3–2.9)	0.834
Pills taken per day		0.040*		0.045*
More than one pill	1		1	
One pill	0.1 (0.0–0.9)		0.1 (0.0–0.9)	
Ever missed pills in the past 2 weeks		0.338		0.311
No	1		1	
Yes	0.6 (0.2–1.6)		0.6 (0.2–1.7)	
Comorbidities		0.504		0.161
No	1		1	
Yes	0.8 (0.4–1.6)		0.5 (0.2–1.3)	

*P < 0.05 is statistically significant. ^&^Crude Odds ratio ^$^Adjusted Odds ratio ^#^95% Confidence Interval.

## Discussion

Most (70%) of the study participants had no prior knowledge about LAI-ART. For those who had prior knowledge, the hospital was their main source of information (65.6%). After educating the patients, 89.6% showed preference for LAI-ART, and the preferred frequency of injections was every 6 months (67.3%). About 41% preferred self-administration of LAI-ART if they are trained to do so and 72.2% were willing to pay for LAI-ART.

Globally, there is a varied level of knowledge of LAI-ART among people living with HIV, most patients are however interested in LAI-ART after education [10–14]. In our study, 30.1% of patients had prior knowledge of LAI-ART, comparable with data from Thailand. This was less than what was obtained in Italy and the US [15–17]. For participants with prior knowledge about LAI-ART before the general education on LAI-ART in our study, about 66% derived their LAI-ART knowledge from hospitals whilst 15% derived it from friends. This is consistent with findings from another study in Connecticut [16].

The willingness to accept LAI-ART in this study was 89.6% which is comparable to what was obtained in France and Italy but twice as high as what was obtained in a US-based study [12,15,18,19]. Similarly, willingness to accept LAI-ART from our study is similar to what was obtained in Uganda, and 7 times more than South Africa [20,21]. In this study, participants who received information about LAI-ART frequently expressed a preference for LAI-ART. However, these findings reflect stated preferences within the study setting and should not be interpreted as evidence that education causes increased acceptance, improved adherence, or better HIV-related outcomes. There should be a cautious interpretation of this finding of high willingness to accept LAI-ART among participants in our study. With the provision of educational knowledge to these participants and assessing their preference after, this may have influenced their responses. Therefore, there is a likelihood of social desirability bias from their responses and therefore this finding reflects a hypothetical preference under controlled informational conditions, rather than an actual behavioral decision in real-world treatment settings. Consequently, the high levels of expressed willingness should be interpreted as indicative of preliminary acceptability rather than a reliable predictor of future uptake.

To achieve viral suppression, over 80–90% adherence to ART is crucial. The medication adherence rate among PLHIV has been reported to be about 77% in Sub Saharan Africa [22–25]. Most (86.7%) of our study participants were adherent on oral ART. This finding was comparable to findings from Cambodia, Nepal, Ethiopia but was higher than what was observed in other studies from Ghana [26–30], Morocco and Egypt [31]. Previous studies have reported associations between differentiated service delivery (DSD) models, retention in care, and adherence outcomes among stable patients on antiretroviral therapy, although causal relationships may vary across settings and study designs [26–31]. While LAI-ART has the potential to improve adherence among some populations, it may simultaneously increase clinic visit frequency compared with multi-month dispensing of oral ART in certain contexts depending on the frequency of administration of the available LAI-ART. The implementation feasibility of LAI-ART in Ghana must be considered within the context of existing differentiated service delivery (DSD) models. Current HIV programmes increasingly rely on multi-month dispensing strategies to reduce clinic attendance, lower patient costs, and decongest health facilities for stable patients on oral ART. In contrast, currently available LAI-ART regimens typically require more frequent clinic visits for administration, monitoring, and follow-up. As a result, injectable ART may complement existing DSD approaches for some patient groups while potentially disrupting established service delivery efficiencies for others. The extent to which LAI-ART integrates effectively into routine HIV care will likely depend on context-specific adaptations, including appointment scheduling systems, workforce capacity, drug storage infrastructure, and mechanisms for decentralized or community-based administration. These structural and health system considerations highlight that patient acceptability alone may not fully predict implementation feasibility or long-term uptake in real-world settings. It is therefore imperative that HIV programmes reflect on how patient preferences for injectable therapy intersect with existing HIV service delivery innovations so as not to complicate the existing implementation activities within the context-specific service delivery system.

In this study, the majority of patients preferred a 6 monthly injection schedule (67.3%), but with the dosing for available LAI-ART being either monthly or bi-monthly, this is not a possibility. Longer dose intervals are generally preferred as seen in this study where 81.6% of patients preferred bi-monthly injections when they were limited to monthly and bi-monthly regimens. This is comparable to data from several studies [12,13,19,32–34]. Although some studies have reported associations between reduced ART dosing frequency and missed appointments, further research is needed to better understand patient experiences and preferences regarding extended-interval injectable therapies such as Lenacapavir [21,35].

Less than half of the patients, 41.1%, preferred self-administration of LAI-ART. This finding was contrary to the finding of over 50% preference for self-administration and out of hospital administration in some studies [15,36]. Previous studies have suggested that these forms of medication administration may be acceptable in some settings and could potentially contribute to reducing clinic congestion, although implementation outcomes are context dependent [36,37].

Despite the high rate of willingness to accept LAI-ART, 32% of patients expressed concerns or fears about LAI-ART side effects, use of needles and its efficacy. Medication efficacy, the cost, fear of needles, possible side effects, and scheduling issues are similar notable concerns raised in other studies [11,12,17–19,33]. These concerns may influence actual uptake and continued use of LAI-ART in routine practice, particularly when combined with health system constraints such as frequent clinic attendance requirements, transportation costs, workforce limitations, and medication supply challenges. Consequently, stated willingness to use LAI-ART in survey settings may differ from real-world treatment adoption and persistence over time. These factors were not explored in our study but are important considerations when switching to LAI-ART from oral ART.

The regression analysis identified an association between being on a once-daily pill regimen and lower reported willingness to accept LAI-ART compared to participants taking more than one pill daily. However, this cross-sectional association should not be interpreted causally and is more appropriately viewed as hypothesis-generating. While LAI-ART might be a good option for a subsection of patients, it might not necessarily be the preferred option for patients depending on the context and health system challenges. Participants on once-daily oral regimens may perceive fewer advantages to switching to LAI-ART, particularly if injectable treatment is perceived to increase clinic attendance requirements. However, actual treatment decisions were not assessed in this study. Thus, it is important that emphasis should be placed on options/informed choices of ART for PLHIV as per the context and health system.

Globally, disruptions in donor support have resulted in service interruptions, staff shortages, weakened supply chains and reduced access to HIV testing and ART. This has resulted in increases in viral load, transmission risk and HIV-related mortality especially where domestic resources cannot compensate for lost aid [5]. LAI-ART implementation involves several operational requirements that could shape patient experiences and reported acceptability in real-world settings. These include the need for regular clinic visits for injections, cold-chain storage infrastructure, trained health personnel to administer injections, and reliable drug procurement systems. And the current disruption in donor support could negatively impact implementation. These programmatic requirements could be associated with differences between stated preferences in survey settings and treatment choices observed during real-world implementation. In addition, health system capacity constraints, such as clinic congestion, workforce limitations, and medication supply stability, may affect both feasibility and patient satisfaction with injectable regimens. Therefore, expressed willingness to accept LAI-ART in survey settings will often differ from real-world treatment adoption, particularly when structural barriers or opportunity costs are introduced.

Our study revealed that about 72% of participants were willing to pay for LAI-ART. This willingness to pay for LAI-ART arises at a time when global HIV donor funding to LMIC is contracting. Modelling studies indicate that cuts to major international programmes such as the President’s Emergency Plan for AIDS Relief (PEPFAR) and Global Fund support could lead to millions of additional HIV infections and deaths [5,38].

Available data reveals a mixed picture as some patients are willing to pay for LAI-ART while others prefer not to pay but rather access LAI-ART via insurance coverage [10,13,17,37,39]. In Ghana, previous studies have reported associations between out-of-pocket payments for HIV-related services and treatment discontinuation among some patients, indicating that even modest co-payments associated with LAI-ART could impose substantial financial strain and undermine adherence if broader funding gaps are not addressed [40].

Reported willingness to contribute financially in this study may not necessarily offset broader systemic donor funding shortfalls, particularly given potential inequities in ability to pay among PLHIV. Finally, health insurance mechanisms, such as the National Health Insurance Scheme in Ghana, can play an important role by pooling risk, reducing out-of-pocket barriers, and facilitating sustainable access to LAI-ART as donor contributions wane, but only if insurance coverage is comprehensive, adequately financed, and integrated with national HIV strategies to absorb the costs of new treatment modalities and protect vulnerable populations [41]. Overall, the findings indicate a high level of reported acceptability of LAI-ART among study participants despite expressed concerns regarding injections, side effects, cost, and efficacy. Higher reported acceptability was observed after participants received information about LAI-ART. However, the study design does not permit conclusions regarding whether awareness or education directly caused increased acceptability. A substantial proportion of participants reported willingness to pay out of pocket for LAI-ART, although stated willingness may differ from actual payment behavior in routine care settings. This study contributes useful preliminary insights into patient perceptions of LAI-ART, highlighting both the promise of long-acting therapies and the substantial implementation challenges that must be addressed before widespread adoption can occur. The study provides preliminary context-specific evidence that may help inform future policy discussions and implementation planning regarding LAI-ART in Ghana. Given the cross-sectional nature of the study, all regression findings should be interpreted as statistical associations rather than causal relationships. The identified associations are useful for generating hypotheses and informing future longitudinal or implementation research.

Despite the strengths of this study, it was conducted in single, urban tertiary healthcare facility among a clinic-based sample and therefore may not fully capture the views, experiences, and preferences of PLHIV in rural or less resourced settings in Ghana. The concept of willingness measured in this study should be interpreted with caution due to the hypothetical nature of the willingness outcome measure and therefore this limits generalizability of the finding. Participants were provided with educational information about LAI-ART prior to reporting their willingness to adopt the therapy, which may have influenced responses through information framing or social desirability bias effects. Consequently, the high levels of expressed willingness should be interpreted as indicative of preliminary acceptability rather than a reliable predictor of future uptake. Additionally, the study focused primarily on patient perspectives and did not assess health system readiness, including infrastructure, workforce capacity, supply chain requirements, and policy preparedness for the implementation of LAI-ART. Additional research employing longitudinal designs, implementation trials, or discrete choice experiments could provide deeper insights into the determinants of patient preferences for LAI-ART in sub-Saharan Africa.

## Conclusion

This study demonstrated that PLHIV in our study setting were generally willing to accept LAI-ART following appropriate education, despite expressed concerns such as cost. Participants showed a preference for longer dosing intervals and administration within hospital settings. These findings reflect preliminary acceptability under informed conditions, rather than demonstrated demand or uptake. However, further implementation research, including longitudinal and real-world studies are needed to comprehensively examine patient-, provider-, and system-level barriers that may influence the uptake and sustained use of LAI-ART among PLHIV.

## Supporting information

S1 Checklist Inclusivity in global research. (DOCX)

S1 Data Data. (XLSX)

S1 Questionnaire Questionnaire for LAI ART (patients). (DOCX)
