# Supplementary material for: Willingness to accept long-acting injectable antiretroviral therapy among persons living with HIV in a tertiary facility in Southern Ghana: Key consideration for programming
Source: PLOS Glob Public Health. 2026 Jun 26;6(6):e0005984. doi: 10.1371/journal.pgph.0005984 (PMC13308768; doi:10.1371/journal.pgph.0005984)
Supplement: S1 Questionnaire — (DOCX) [file pgph.0005984.s003.docx]

## QUESTIONNAIRE FOR LAI ART (PATIENTS)

**SOCIO- DEMOGRAPHIC CHARACTERISTICS**

1. FACILITY………………….. STUDY ID ………………
2. SEX A. FEMALE……….. B. MALE…………..
3. AGE (YEARS)……………………
4. MARITAL STATUS A. SINGLE……….. B. MARRIED…….. C. SEPARATED…… D. DIVORCED….. E. WIDOWED……..
5. RELIGION A.CHRISTIANITY..B. ISLAM…C.TRADITIONAL…

D.OTHER..

1. HIGHEST EDUCATION A. NONE…B. PRIMARY…C. JHS…

D. MSLC…………… E. SHS……….F. VOC/ TECH………

G. TERTIARY………..

1. OCCUPATION A. NONE………B. STUDENT……C. EMPLOYED………

D. SELF- EMPLOYED…. E. RETIRED……..

1. PLACE OF RESIDENCE………………..

**CLINICAL CHARACTERISTICS**

1. YEAR OF DIAGNOSIS ……………… 1I. NUMBER OF YEARS SINCE HIV DIAGNOSIS………………
2. YEAR OF START OF ART………. 2I. NUMBER OF YEARS ON ART……………….
3. CURRENT ART REGIMEN (TICK AS MANY AS APPLICABLE)
4. TENOFOVIR……. B. ABACAVIR……….. C. ZIDOVUDINE……… D. LAMIVUDINE …….

E. EMTRICITABINE….. F. EFAVIRENZ……. G. NEVIRAPINE……. H. DOLUTEGRAVIR…

I. ATAZANAVIR/ RITONAVIR……… J. LOPINAVIR/ RITONAVIR…… K. OTHERS (please state)

1. NUMBER OF ART PILLS TAKEN PER DAY…………
2. HAVE YOU EVER MISSED AN ART PILL IN THE PAST 2 WEEKS? A. YES…… B. NO…………..

IF YES, GO TO QUESTION 5I AND 5II.

5I. HOW MANY PILLS DID YOU MISS?....................

5II. REASON FOR MISSING PILL(S).

1. RAN OUT OF PILLS….. B. WAS TOO ILL… C. TAKING TOO MANY PILLS…..
2. FORGOT.. E. SIDE EFFECTS…. F. FELT DEPRESSED/ ANXIOUS…..
3. TOO BUSY TO TAKE…. H. DIDN’T JUST WANT TO TAKE……..
4. SHARED PILLS WITH OTHERS….. J. I WAS WELL, SO NO NEED FOR PILLS…….
5. OTHERS…………………………………………………….
6. CO- MORBIDITIES
7. HYPERTENSION…B. DIABETES ……C. DYSLIPIDAEMIA…
8. CKD…E. CLD…… F. OTHERS…
9. ARE YOU ON ANY OTHER MEDICATIONS OTHER THAN ART? A. YES………… B. NO………

IF YES, GO TO 7I.

7I. WHAT OTHER MEDICATIONS ARE YOU ON?

1. …………………………
2. ………………………..
3. …………………………
4. …………………………

**KNOWLEDGE AND PERCEPTION OF** **LONG- ACTING INJECTABLE ART**

1. Do you know of any other form in which you can take your ARVs aside swallowing tablets?

A.Yes B.No

1. If yes, kindly state which other forms you know of ……………………………………..
2. Which forms would you prefer? (Multiple choice but rank them from your best to worse)
3. Swallowing pills only
4. Injections only
5. Mix of swallowing and injections
6. Have you heard of Long- Acting Injectable ART?
7. YES……… B. NO…………..

4A.If yes, where did you hear it from? A. Hospital/clinic B. Friends C. Radio D. Television E. Social media (facebook/twitter/Instagram etc)

4B If no, research assistant to explain what LAIs are. Then continue with the questionnaire administration.

1. Would you use Long- Acting Injectable ART?
2. Yes……… B. No…….. C. I don’t know………….

5A.If yes,

How often do you want the injections for the LAI to be administered?

Once a month

Once every 2 months

Once every 3 months

Once every 6 months

1. The types of LAI currently available are once a month and once every 2 months. Which one will you prefer?
2. Once a month
3. Once every 2 months
4. Who would you prefer to give you the injection
5. A trained nurse
6. A trained volunteer
7. Would you prefer the option of being trained to give the injection yourself at home just like it is done for those with diabetes?
8. YES…….. B. NO……….. C. Indifferent
9. Would you consider paying for LAI?
10. YES……… B. NO…………. C. I DON’T KNOW……………
11. Do you have any concerns/fears about use of long-acting injectables? A. Yes B. No
